# Validation of the Japanese version of the Body Image Scale for bladder cancer patients

**DOI:** 10.1038/s41598-022-25669-2

**Published:** 2022-12-13

**Authors:** Miho Sato, Takahiro Osawa, Takashige Abe, Michitaka Honda, Madoka Higuchi, Shuhei Yamada, Jun Furumido, Hiroshi Kikuchi, Ryuji Matsumoto, Yasuyuki Sato, Yoshihiro Sasaki, Toru Harabayashi, Satoru Maruyama, Norikata Takada, Keita Minami, Hiroshi Tanaka, Ken Morita, Akira Kashiwagi, Sachiyo Murai, Yoichi M. Ito, Katsuhiko Ogasawara, Nobuo Shinohara

**Affiliations:** 1 Faculty of Health Sciences, Hokkaido University, Sapporo, Japan; 2 Department of Urology, Hokkaido University Graduate School of Medicine, N15 W7 Kita-ku, Sapporo, 060-8638 Japan; 3 Department Minimally Invasive Surgical and Medical Oncology, Fukushima Medical University, Fukushima, Japan; 4 Department of Urology, Keiyukai Hospital, Sapporo, Japan; 5 Department of Urology, Kushiro Rosai Hospital, Kushiro, Japan; 6 Department of Urology, Hokkaido Cancer Center, Sapporo, Japan; 7 Department of Urology, Sapporo City General Hospital, Sapporo, Japan; 8 Department of Urology, Kushiro City General Hospital, Kushiro, Japan; 9 Department of Urology, Teine Keijinkai Hospital, Sapporo, Japan; 10 Clinical Research and Medical Innovation Center, Hokkaido University Hospital, Sapporo, Japan

**Keywords:** Surgical oncology, Bladder cancer, Urological manifestations, Urological cancer

## Abstract

The Body Image Scale (BIS) is a 10-item tool that measures the body images of cancer patients. This study aims to validate the Japanese version of the BIS for bladder cancer patients. A multicenter cross-sectional survey was used to identify the participants, which included Japanese bladder cancer patients. The percentage of missing responses, internal consistency, and known-group validity were evaluated. The correlations between the BIS and two HRQOL instruments (the Bladder Cancer Index and the SF-12) were assessed to determine convergent validity. Among 397 patients, 221 patients were treated by transurethral resection of bladder tumor (TURBT) endoscopically, 49 patients underwent cystectomy with neobladder, and 127 patients underwent cystectomy involving stoma. The percentage of missing responses in the BIS ranged from 8.1 to 15.6%. Cronbach's α coefficient was 0.924. Higher BIS scores indicate negative body image, and the median BIS score for patients with native bladders after TURBT (0.5) was significantly lower than those of the patients with neobladder (4.0) and stoma formation (7.0), which indicated the discriminatory ability of the BIS. Each domain of the Bladder Cancer Index and the role summary score of the SF-12 correlated to the BIS scores, which confirmed the convergent validity. A range of BIS scores were identified among patients who reported similar physical summary scores and mental summary scores of the SF-12. This study confirmed the reliability and validity of the Japanese version of the BIS for bladder cancer patients.

## Introduction

Cancer and its treatment often affect the body in several ways, such as physical damage, appearance changes or functional impairments. These changes can negatively affect an individual’s perception of his or her body^1^. Many studies have shown that lower body image is associated with negative psychosocial consequences in cancer patients, including those with breast cancers, head and neck cancers, and prostate cancer^2–4^. Body image has recently become an important component of health-related quality of life (HRQOL) in cancer patients.

Bladder cancer is a common malignancy of the urinary tract, and the incidence is higher in men than in women^5^. Transurethral resection of bladder tumor (TURBT) is an endoscopic surgery. After TURBT, patients maintain their native bladder and do not have any change in their appearance. On the other hand, radical cystectomy, in which the native bladder is removed, often impacts patients’ lives due to fundamental changes in urinary function, and it can lead to appearance changes accompanied by surgical scars and stoma formation, resulting in disturbances to body image. Patients with stoma are more likely to experience alteration of body appearance. Therefore, body image concerns need to be assessed and managed in a timely manner, and a valid and reliable measurement tool is needed.

The Body Image Scale (BIS) is a tool to assess body image concerns in patients with cancer. It is a patient-reported outcome measure that was developed by Hopwood et al.^6^ in collaboration with the European Organization for Research and Treatment of Cancer (EORTC) Quality of Life Study Group. The BIS is a widely used measurement, and the original version has been translated into several languages, such as Spanish, Italian, Dutch and Korean, with good psychometric properties^7–10^. The BIS is a useful tool in oncology research because it has been developed to apply to all types of cancer patients regardless of the type of treatment that the patient receives. For these reasons, using the BIS has benefits, but there are no reports on the Japanese version. Therefore, this study aims to investigate the reliability and validity of the newly developed Japanese version of the BIS in a multicenter population of bladder cancer patients in Japan.

## Methods

### Participants

This was a cross-sectional observational study. The sample consisted of 450 bladder cancer patients from 7 institutions in Japan. Responses were obtained from 397 patients. The inclusion criteria were as follows: patients older than 20 years of age, with an Eastern Cooperative Oncology Group performance status (ECOG-PS) of 0 or 1. The study protocol was approved by each institutional review board, and the approval number for Hokkaido University Hospital was 015-0504. All patients provided written informed consent. The methods were carried out in accordance with the approved guidelines. All procedures performed in the studies were in accordance with the 1964 Helsinki declaration.

### Measures

The BIS is a self-reported 10-item scale that evaluates the cognitive, affective and behavioral aspects of body image among cancer patients^6^. The responses are measured on a four-point scale (not at all = 0, a little = 1, quite a bit = 2, and very much = 3). The total scores, calculated by summing the 10 items, ranged from 0 to 30. Higher scores indicated a more negative body image. The Japanese version of the BIS was developed with permission from the developer, following the standard procedure of forward and back translation. Two Japanese translations of the BIS were carried out independently by two native Japanese physicians who had English as a second language. Subsequently, the reconciled translation was back-translated into English by a native English professional translator. Of the 10 items in the BCI, 4 overlapped with the QLQ-BR23, and therefore, the Japanese version of the QLQ-BR23^11^ was applied with the permission of Dr. Kojiro Shimozuma. The two forms of the BIS (the original and the backward translation) were compared, discussed, and resolved in a consensus meeting, resulting in the draft Japanese BIS version. This draft version was then reviewed by laypersons fluent in Japanese to develop a linguistically valid, easy-to-understand Japanese version.

To investigate convergent validity, two psychometrically validated HRQOL instruments, the Bladder Cancer Index (BCI)^12^ and the SF-12^13^, were applied. The BCI is a bladder cancer-specific HRQOL instrument. It assesses symptom magnitude and impairment in domains of urinary, bowel and sexual health. Higher scores indicate better health status. The SF-12 is a generic HRQOL measure that has been used across a variety of health conditions. We calculate the physical, mental and role component summary scores. The patients’ sociodemographic and clinical data were obtained from physicians.

### Statistical analysis

The percentage of missing responses was calculated for each item. The profiles of the responders and nonresponders were compared in terms of age, sex and treatment type using the Mann–Whitney U test or chi-square test. The total score was calculated for participants who responded to 9 items out of 10 items. When one item was missing, the score was calculated by imputing the mean score of the nonmissing data. For the total score, the percentages of lowest score (0) and highest score (30) were calculated to evaluate the floor and ceiling effects according to treatment groups.

Factor structure was examined using an exploratory factor analysis with the generalized least squares method. The Kaiser-Guttman criteria with an eigenvalue greater than 1 determined the number of factors. The internal consistency was examined by calculating Cronbach’s alpha (α) coefficients.

To evaluate known-group validity, we compared the BIS scores with the Bonferroni-adjusted Mann–Whitney U test among the following treatment groups: (1) patients who underwent TURBT endoscopically, (2) patients who underwent cystectomy involving ileal neobladder diversion, and (3) patients who underwent radical cystectomy involving ileal conduit diversion or cutaneous ureterostomy accompanied by stoma formation. Cutaneous ureterostomy was generally reserved for elderly patients with a limited life expectancy and patients with severe comorbidities. Particularly in a patient with a single kidney, cutaneous ureterostomy was preferred and was performed on the ipsilateral site. We also compared each domain score of the BCI and each summary score of the SF-12 according to these treatment groups as a reference of the discriminability of the other HRQOL instruments. Correlation analysis was performed, and the scatter plots with 95% confidence interval ellipses were described to show data clustering.

Data analyses were performed using IBM^®^ SPSS^®^ Statistics V22.0 (IBM, Chicago, Illinois, USA) and JMP Pro 16 (SAS Institute Inc., Cary, NC, USA), and a p value of < 0.05 was considered statistically significant.

## Results

### Participant characteristics

Table 1 shows the sociodemographic and clinical characteristics of the sample. The median age was 72 years, and 301 participants (75.8%) were male. Two hundred and twenty-one patients (55.7%) had native bladder after TURBT, 49 patients (12.3%) underwent cystectomy with neobladder, 101 patients (25.4%) were treated by cystectomy involving ileal conduit diversion, and 26 patients (6.5%) were treated by cystectomy involving cutaneous ureterostomy.

**Table 1:** Characteristics of the sample.

	No (%)
Median age (IQR)	72 (65–77)
**Sex**	
Male	301 (75.8)
Female	96 (24.1)
**Treatment**	
Transurethral resection of bladder tumor	221 (55.7)
Cystectomy, neobladder	49 (12.3)
Cystectomy, stoma	
Cystectomy, ileal conduit diversion	101 (25.4)
Cystectomy, cutaneous ureterostomy	26 (6.5)
**ECOG performance status**	
0	362 (91.2)
1	27 (6.8)
Unknown	8 (2.0)
**Stage**	
Ta, Tis, T1	294 (74.1)
T2–T4	95 (23.9)
Unknown	8 (2.0)
CIS present	71 (17.9)
**Grade**	
Low	155 (39.0)
High	223 (56.2)
Unknown	19 (4.8)
**Histology**	
UC	367 (92.4)
Other	30 (7.6)
Months since surgery (IQR)	29 (12–66)

### Feasibility

The percentage of missing responses ranged from 8.1 to 15.6% in the full sample, and only item 10 exhibited a missing response rate of ≥ 15%. Compared with responders, nonresponders were older, more likely to be female than male and more likely to have native bladder after TURBT than to have undergone cystectomy (Supplementary Table S1).

The patients who had the highest score (30) were not reported in any treatment groups. The percentages of patients who had the lowest score (0) were patients with native bladder after TURBT (50%), patients who underwent cystectomy with neobladder (15.2%) and patients who underwent cystectomy with stoma (15.9%). When describing the responses for each item of the BIS, the percentages of patients who reported a score of 0 were highest among those with native bladder after TURBT on most of items (Supplementary Fig. S1).

### Factor analysis and internal consistency

The results of the exploratory factor analysis determined that all items for each sample loaded on a single factor of 0.649–0.835, which explained 57.07% of the variance. Cronbach’s α coefficient was 0.924 (Supplementary Table S2).

### Comparison among treatment groups

**Figure 1:**
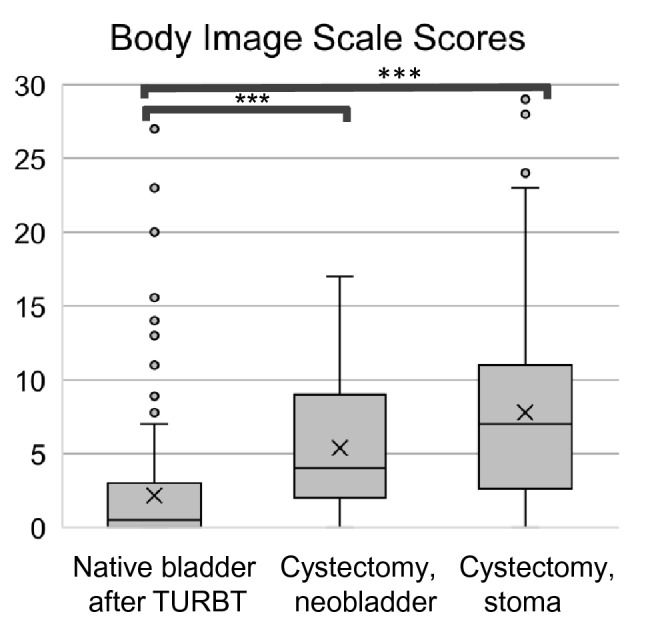
Known group validity: Body Image Scale (BIS) scores by treatment groups. Bonferroni adjusted Mann–Whitney U test was used to compare among treatment groups (***p < 0.001).

Known-group validity was assessed by comparing BIS scores among the three patient groups according to treatment. Figure 1 shows a boxplot with the median and distribution of BIS scores by treatment group. The medians of BIS were 0.5 (IQR: 0–3.0) in patients with native bladder after TURBT, 4.0 (IQR: 2.0–9.0) in patients undergoing cystectomy with neobladder, and 7.0 (IQR: 2.6–11.0) in patients undergoing cystectomy with stoma. The patients with native bladder after TURBT showed significantly lower BIS scores, indicating that they had better body images than patients undergoing cystectomy with neobladder and stoma (p < 0.001 for both comparisons).

Regarding the urinary domain scores of BCI, there were significant differences among the three treatment groups (p < 0.001 for all comparisons). The bowel domain scores of the BCI were also significantly different between patients undergoing native bladder after TURBT and patients undergoing cystectomy with neobladder (p < 0.001), between patients undergoing native bladder after TURBT and patients undergoing cystectomy with stoma (p < 0.01), and between patients undergoing cystectomy with neobladder and patients undergoing cystectomy with stoma (p < 0.05). Regarding sexual domain scores, patients with neobladders or stomata had lower scores than patients with native bladders after TURBT (p < 0.001 for both comparisons). Regarding each summary score in the SF-12, patients with stoma groups had lower role summary scores than patients with native bladder after TURBT (p < 0.001), but physical and mental summary scores were not differentiated by treatment (Supplementary Fig. S2).

### Correlation analysis

**Figure 2:**
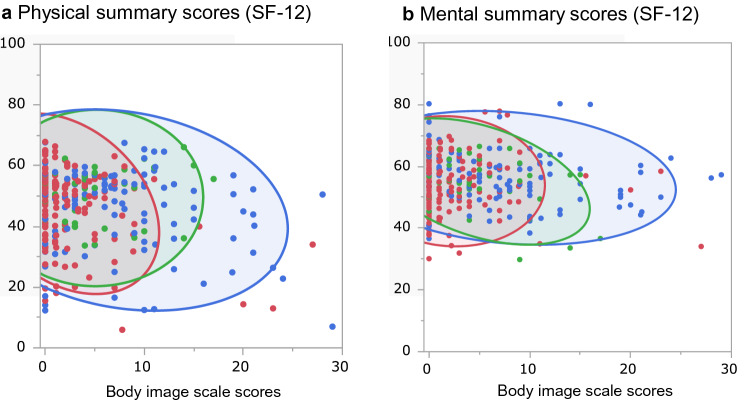
Scatterplots of the Body Image Scale (BIS) scores and the physical and mental summary scores of the SF-12. The 95% confidence interval ellipses show the endoscopy treatment group (red), neobladder group (green), and stoma group (blue) in each figure.

The correlation coefficients between the BIS and each domain score of the BCI and each summary score of the SF-12 were − 0.536 for the urinary domain, − 0.361 for the bowel domain, − 0.323 for the sexual domain, − 0.109 for the physical summary score, − 0.088 for the mental summary score, and − 0.406 for the role summary score (Supplementary Table S3). The relationship among the BIS and the physical summary score and mental summary score of the SF-12 is shown in Fig. 2. Encompassed by 95% confidence interval ellipses, the plots describe a horizontal ellipse in the direction of the x-axis in scatterplots. It demonstrated a range of BIS scores even among patients who reported physical and mental summary scores at the same level.

## Discussion

This is the first study to evaluate psychometric properties of the Japanese version of the BIS in bladder cancer patients via a multicenter cross-sectional survey. Our results demonstrate the reliability and validity of the Japanese version of the BIS.

The first major finding in this study is that the Japanese version of the BIS has a one-factor structure, as shown in the original BIS. Moreover, the Japanese version of the BIS has a high Cronbach’s alpha coefficient, which is equivalent to previous studies (0.91–0.96)^6–9^. This indicates that the Japanese version of the BIS has sufficient internal consistency reliability, as shown in the original BIS.

The BIS scores of both the patients with neobladder and those with stoma were significantly worse than those with native bladder who had undergone endoscopic treatment. This is reasonable because endoscopic treatment involves transurethral resection of the bladder and does not result in appearance changes accompanying stoma or surgical scars on the abdomen. A similar finding was reported in patients with colorectal cancer with stoma^14^. This result supported the discriminatory ability of the BIS. Although the difference did not reach statistical significance, the patients with neobladders had better body images than those with stomata in this study. Since stoma is more likely to cause alteration of body appearance, it is a reasonable result. Comparable findings were previously reported by Clements et al.^15^, showing that the body images of patients with continent diversion were better than those with ileal conduit diversion in longitudinal data of 411 cohorts. Most likely, our sample size undermined the statistical power to detect the difference.

The effects of age and gender on body image among cancer patients were described in previous articles. Younger patients who received treatments for breast cancer or head and neck cancer are more likely to experience body image dissatisfaction^3, 16^. Women tend to experience worse body image distress in colorectal cancer patients^17^. The current study could not fully evaluate the effects of age and gender on body image, because bladder cancer mostly affects older males, as in the population of the current study.

Convergent validity was demonstrated by the correlation between the BIS and each domain of the BCI and BIS and the role summary component of the SF-12. The analysis showed that the patients with worse BCI scores and those with worse role function tended to have worse body image scores. Previous literature explained that the construct of body image is not limited to changes in outward appearances but also to changes in self-image^18^, and these changes are related to disfigurement and dysfunction^19^. In the current study, when daily living functions such as urinary, bowel and sexual function were negatively altered, the perception of the body was likely to be negatively changed, leading to worse body image.

The scatterplots of the BIS and the physical summary score and mental summary score of the SF-12 took the shape of a horizontal ellipse, indicating a range of BIS scores even among patients who reported physical and mental summary scores at the same level. This suggests that the BIS detects HRQOL-specific body image deterioration that the SF-12 cannot. Thus, BIS more specifically identifies symptoms related to body image after bladder cancer treatment.

The results regarding the missing responses reflected the acceptable feasibility of BIS. We found that the missing values were associated with age, sex and treatment type. Therefore, the BIS data obtained from older patients, female patients and patients with native bladder after TURBT should be interpreted with some caution.

The percentage of patients who reported the lowest score (0) was highest among patients with native bladder after TURBT but low in patients with cystectomy, which is an acceptable result. No ceiling effect was observed in any treatment group.

Considering which items are more relevant to bladder cancer is an interesting area of research. For example, in the case of breast cancer, four items related to body image are included in the EORTC Breast Cancer Module, the QLQ-BR23^20^. Items 1, 2, 5, and 7 in the BIS might be considered candidates to distinguish body images between patients who underwent cystectomy with a neobladder and those who underwent cystectomy with a stoma, since the percentages of subjects who responded with scores > 0 differed between the treatment groups (Supplementary Fig. S1).

We compared the BCI and the SF-12 according to three treatment groups as a reference of the discriminability. The results provided valuable data for understanding the postoperative HRQOL for bladder cancer patients, since there are few reports on this topic. Regarding bladder cancer-specific HRQOL measured by BCI, we found lower urinary and bowel scores for patients with neobladder than for the other two groups. This result is consistent with previous work^21^. One of the reasons for this is supposed to be the unexpected incontinence or the occasional need for urethral catheterization. These sequalae are sometimes inevitable after cystectomy with neobladder. In addition, the bowel domain score for the patients after cystectomy was lower than that for those not undergoing cystectomy. This suggests that trouble related to bowel movement was more frequent in patients who underwent neobladder or stoma than in those who did not undergo cystectomy. The analysis of SF-12 scores according to each treatment group clarified that the role summary score of patients with stoma was significantly lower than that of patients with native bladder after TURBT. It possibly relies on the challenges of daily and social activities, such as the complexity of managing the stoma, especially away from home^22^. However, there was no difference in the physical summary score or mental summary score among the three treatment groups.

There were several limitations of this study. This study was a cross-sectional study, so we did not examine the test–retest reliability. Additionally, body image may change over time. Longitudinal evaluation is desirable in the future. Since the subjects in this study were not randomly sampled, the possibility of selection bias might have been unavoidable. The sample was not large enough to validate the known-group analysis based on age and gender. Last, the BIS includes 10 questions, which is a relatively long questionnaire to respond to in clinical settings.

Despite the limitations, this is the first study to validate the Japanese version of the BIS in a sample using a multicenter population of bladder cancer patients in Japan. The BIS has sufficient internal consistency reliability. The BIS has discriminatory ability and captures the different aspects of HRQOL that are not identified by the generic HRQOL. The use of the Japanese version of the BIS contributes to the evaluation of patient-reported outcomes with respect to HRQOL. The validation of BIS for diverse groups of cancer patients is desirable and will provide an opportunity to identify effective interventions that promote body image in research and clinical settings.

## Supplementary Information

Supplementary Information.

## Data Availability

All data analyzed during the study are included in this article.

## References

[1] Fingeret MC, Teo I, Epner DE (2014). Managing body image difficulties of adult cancer patients: Lessons from available research. Cancer.

[2] Kołodziejczyk A, Pawłowski T (2019). Negative body image in breast cancer patients. Adv. Clin. Exp. Med..

[3] Fingeret MC (2012). The nature and extent of body image concerns among surgically treated patients with head and neck cancer. Psychooncology.

[4] Taylor-Ford M (2013). Body image predicts quality of life in men with prostate cancer. Psychooncology.

[5] Matsuda T, Okuyama A (2017). Incidence rate for bladder cancer in Japanese in Japan and in the United States from the Cancer Incidence in Five Continents. Jpn. J. Clin. Oncol..

[6] Hopwood P, Fletcher I, Lee A, Al Ghazal S (2001). A body image scale for use with cancer patients. Eur. J. Cancer.

[7] Gómez-Campelo P, Bragado-Álvarez C, Hernández-Lloreda MJ, Sánchez-Bernardos ML (2015). The Spanish version of the body image scale (S-BIS): Psychometric properties in a sample of breast and gynaecological cancer patients. Support Care Cancer.

[8] Annunziata MA (2018). A contribution to the validation of the Italian version of the Body Image Scale (BIS). BMC Cancer.

[9] van Verschuer VM, Vrijland WW, Mares-Engelberts I, Klem TM (2015). Reliability and validity of the Dutch-translated body image scale. Qual. Life Res..

[10] Khang D, Rim HD, Woo J (2013). The Korean version of the body image scale-reliability and validity in a sample of breast cancer patients. Psychiatry Investig..

[11] Shimozuma K (2000). Reliability and validity of the Japanese version of the Functional Assessment of Cancer Therapy-Breast (FACT-B) quality-of-life instrument; Women's Health Outcome Study (WHOS)-01. Quality Life Res..

[12] Osawa T (2019). Development of the Japanese version of the health-related quality of life questionnaire for bladder cancer patients using the bladder cancer index: A pilot study. Int. J. Urol..

[13] Ware J, Kosinski M, Keller SD (1996). A 12-item short-form health survey: Construction of scales and preliminary tests of reliability and validity. Med. Care.

[14] Whistance RN (2010). Assessment of body image in patients undergoing surgery for colorectal cancer. Int. J. Colorectal Dis..

[15] Clements MB (2022). Health-related quality of life for patients undergoing radical cystectomy: Results of a large prospective cohort. Eur. Urol..

[16] Chen CL, Liao MN, Chen SC, Chan PL, Chen SC (2012). Body image and its predictors in breast cancer patients receiving surgery. Cancer Nurs..

[17] Reese JB, Handorf E, Haythornthwaite JA (2018). Sexual quality of life, body image distress, and psychosocial outcomes in colorectal cancer: A longitudinal study. Support Care Cancer.

[18] Hopwood P (1993). The assessment of body image in cancer patients. Eur. J. Cancer.

[19] Rhoten BA, Murphy B, Ridner SH (2013). Body image in patients with head and neck cancer: A review of the literature. Oral Oncol..

[20] Sprangers MA (1996). The European organization for research and treatment of cancer breast cancer-specific quality-of-life questionnaire module: First results from a three-country field study. J. Clin. Oncol..

[21] Osawa T (2020). Health-related quality of life in Japanese patients with bladder cancer measured by a newly developed Japanese version of the bladder cancer index. Int. J. Clin. Oncol..

[22] Villa G (2018). Life with a urostomy: A phenomenological study. Appl. Nurs. Res..

