# Supplementary material for: Validation of the Japanese version of the Body Image Scale for bladder cancer patients
Source: Sci Rep. 2022 Dec 13;12:21544. doi: 10.1038/s41598-022-25669-2 (PMC9747706; doi:10.1038/s41598-022-25669-2)
Supplement: Supplementary file 1 — Supplementary Information. [file 41598_2022_25669_MOESM1_ESM.pdf]

Supplementary Table S1 Missing values of each item and comparison between responders and nonresponders

	Missing Values (%)	Age *p* value (Mann-Whitney U Test)	Sex *p* value (chi-square test)	Treatment *p* value (chi-square test)
Self-conscious	8.1	<0.001	<0.001	0.502
Less physically attractive	8.6	<0.001	<0.001	0.484
Dissatisfied with appearance	9.3	<0.001	0.001	0.895
Less feminine or less masculine	9.1	<0.001	<0.001	0.696
Difficult to see self naked	11.1	0.033	0.002	0.223
Less sexually attractive	9.8	<0.001	<0.001	0.442
Avoid people	11.1	<0.001	<0.001	0.247
Body less whole	8.1	<0.001	0.007	0.502
Dissatisfied with body	9.3	0.001	0.004	0.695
Dissatisfied with scar	15.6	0.097	0.010	<0.001

Supplementary Table S2 Factor analysis and internal consistency of the Body Image Scale

	Factor loadings	Cronbachs alpha
Self-conscious	0.689	0.924
Less physically attractive	0.822	
Dissatisfied with appearance	0.762	
Less feminine or less masculine	0.726	
Difficult to see self naked	0.804	
Less sexually attractive	0.737	
Avoid people	0.649	
Body less whole	0.813	
Dissatisfied with body	0.835	
Dissatisfied with scar	0.693	
	% variance<br>57.07	

Supplementary Table S3 Correlation analysis between the Body Image Scale (BIS) and each domain score of the Bladder Cancer Index (BCI) and each summary score of the SF-12

		BIS
BCI	Urinary domain	-0.536
	Bowel domain	-0.361
	Sexual domain	-0.323
SF-12	Physical summary score	-0.109
	Mental summary score	-0.088
	Role summary score	-0.406

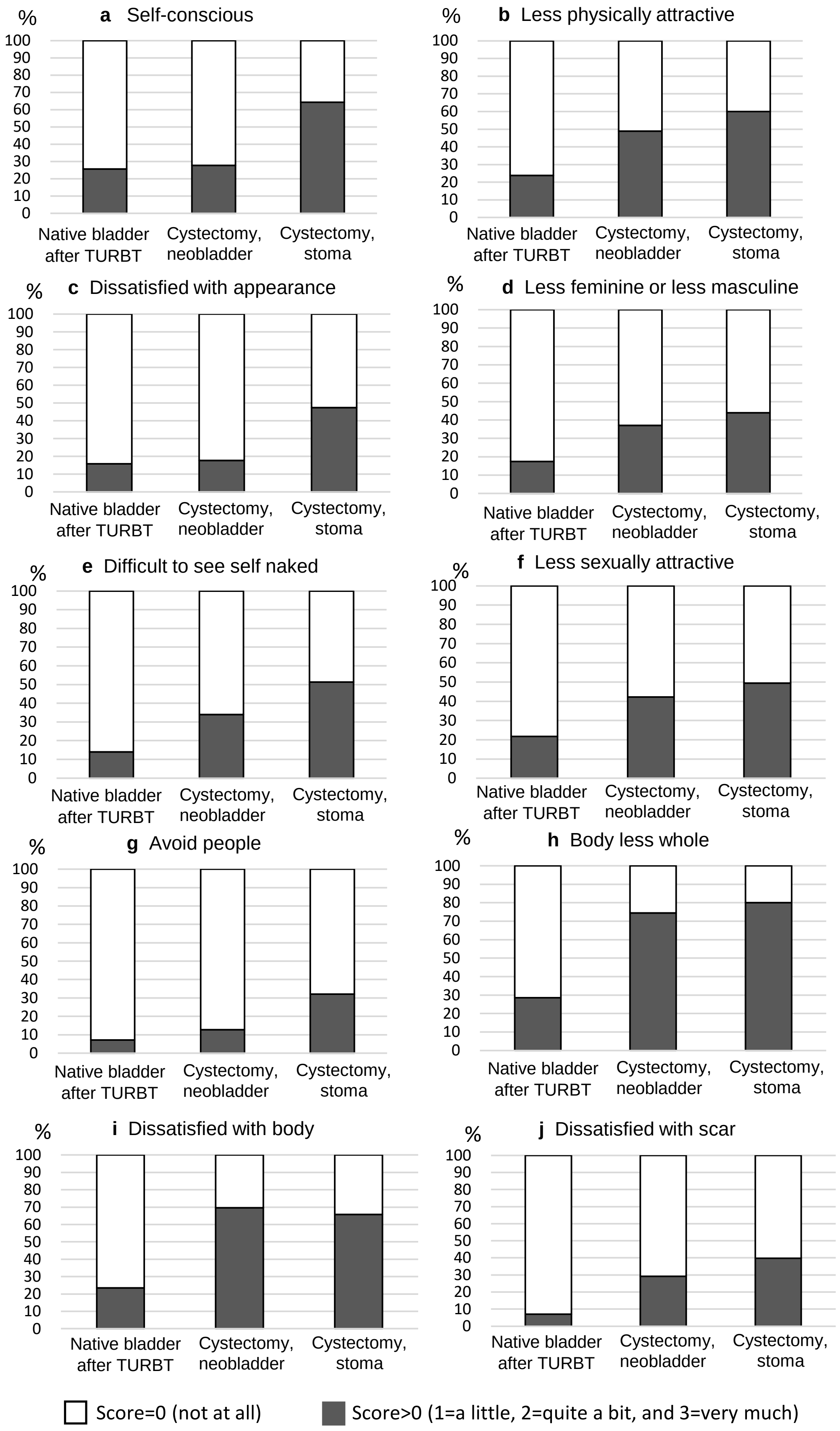

Supplementary Figure S1 The percentage of subjects who responded each items of the Body Image Scale (BIS). Native bladder after TURBT: n=221, cystectomy with neobladder: n=49, cystectomy with stoma n=127).

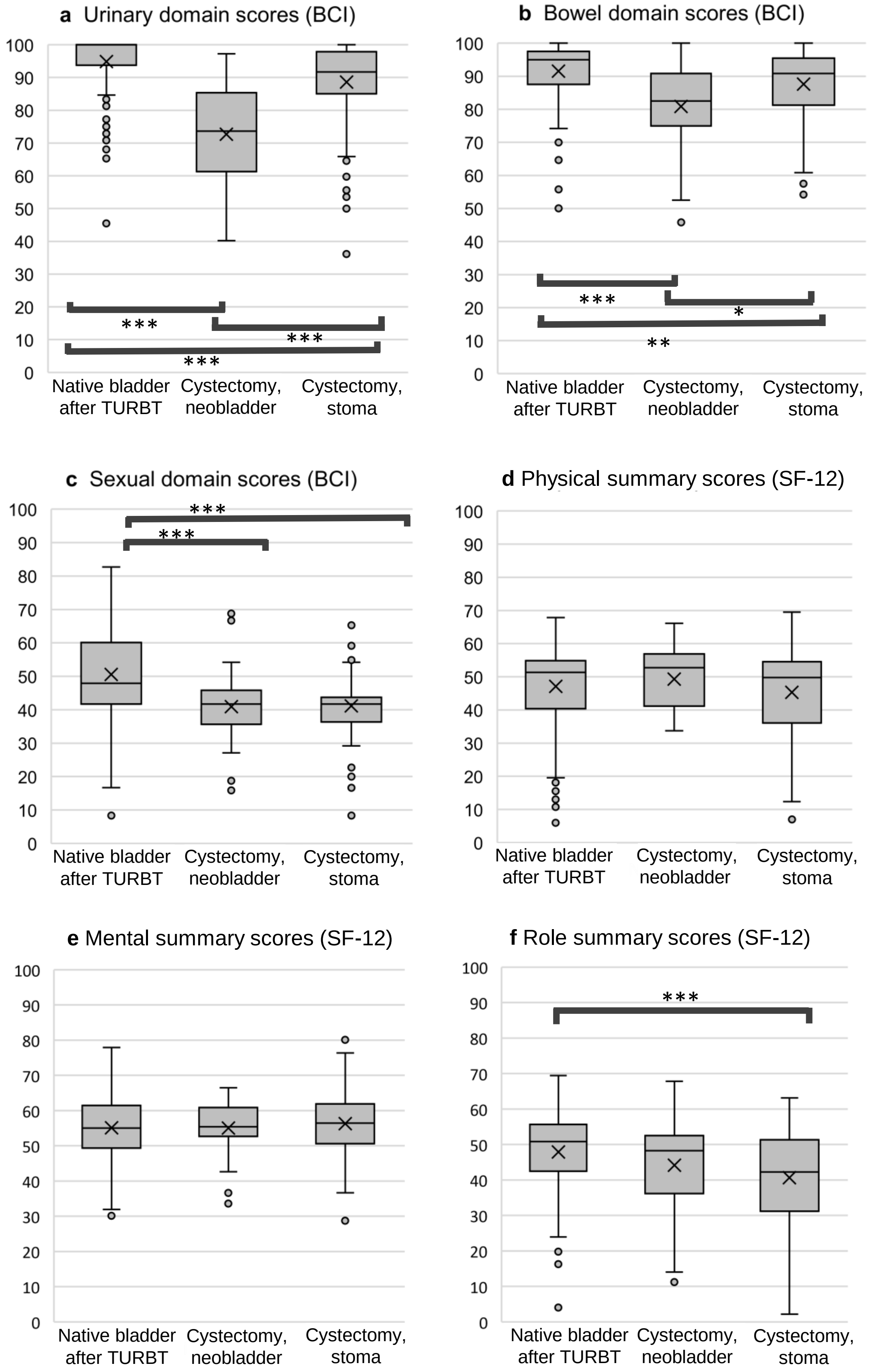

Supplementary Figure S2 Bladder Cancer Index (BCI) domain scores and SF-12 summary scores by treatment groups. Bonferroni adjusted Mann-Whitney U test was used to compare among treatment groups (*p<0.05, **p<0.01, ***p<0.001)

あなたの外見についての満足度および、これまでの病気もしくはその治療により見られた 変化についておうかがいします。**過去 4 週間**についてお考え下さい。

In this questionnaire you will be asked how you feel about your appearance, and about any changes that may have resulted from your disease or treatment. Please reply which comes closest to the way you have been feeling about yourself, during the past month.

	全くない<br>Not at all	少しある<br>A little	多い<br>Quite a bit	とても多い<br>Very much
1） 自分の外見について自意識してしまいましたか。<br>Have you been feeling self-conscious about your appearance?	0	1	2	3
2） 病気やその治療のせいで、身体的な魅力が減ったと感じましたか。<br>Have you felt less physically attractive as a result of your disease or treatment?	0	1	2	3
3） 服を着たときの自分の外見に不満がありましたか。<br>Have you been dissatisfied with your appearance when dressed?	0	1	2	3
4） 病気やその治療のせいで、自分の女性らしさ、または男性らしさがあまりなくなったと感じましたか。<br>Have you been feeling less feminine/masculine as a result of your disease or treatment?	0	1	2	3
5） 自分の裸を見るのがつらかったですか。<br>Did you find it difficult to look at yourself naked?	0	1	2	3
6） 病気やその治療のせいで、自分の性的な魅力が減ったと感じましたか。<br>Have you been feeling less sexually attractive as a result of your disease or treatment?	0	1	2	3
7） 自分の外見が気になるので、人と会うことを避けましたか<br>Did you avoid people because of the way you felt about your appearance?	0	1	2	3
8） 病気やその治療により、自分の体が完全なものではなくなったと感じましたか。<br>Have you been feeling the treatment has left your body less whole?	0	1	2	3
9） 自分の身体について不満がありましたか。<br>Have you felt dissatisfied with your body?	0	1	2	3
10） （手術を受けた場合にのみお答え下さい。）<br>自分の手術の傷について不満がありましたかたか。<br>(If applicable)<br>Have you been dissatisfied with the appearance of your scar?	0	1	2	3

Supplementary Figure S3 Japanese version of Body Image Scale
